# Case report: Gastric langerhans cell histiocytosis with *BRAF* deletion mutation in a child that was misdiagnosed as lymphoma

**DOI:** 10.3389/fonc.2024.1443553

**Published:** 2025-01-22

**Authors:** Zhi Wan, Xue Tang, Ju Gao, Jing-jing Sun

**Affiliations:** ^1^ Department of Pediatrics, West China Second University Hospital, Sichuan University, Chengdu, China; ^2^ Key Laboratory of Birth Defects and Related Diseases of Women and Children, Sichuan University, Ministry of Education, Chengdu, China

**Keywords:** langerhans cell histiocytosis, stomach, *BRAF*, child, lymphoma

## Abstract

Langerhans cell histiocytosis (LCH) is a myeloid neoplasm associated with the infiltration of most organs but rarely involves the stomach. Stomach tumors in children are very rare and can be easily misdiagnosed. We report the first case of gastric LCH in a 9-year-old boy who was misdiagnosed with gastric lymphoma. The patient presented to our outpatient department with recurrent abdominal pain that had lasted 1 month. Due to the absence of typical clinical features associated with lymphoma in the boy, the initial diagnosis of lymphoma based on the first gastric pathological biopsy was questioned. The second pathological examination revealed that the tumor cells expressed CD1a, S-100, and Langerin with *BRAF* (c.1457_1471del) deletion mutations. The patient’s condition rapidly improved after chemotherapy with prednisone and vincristine. This case report focuses on the possibility of gastric LCH in school-aged children and the differential diagnosis of gastric tumors in children.

## 1 Introduction

Langerhans cell histiocytosis (LCH) is a myeloid neoplasm of mixed cellularity, which is characterized by activating mutations in the mitogen-activated protein kinase (MAPK) pathway (1). LCH occurs prevalently but not exclusively in children, with an incidence of 3 to 9 cases per million children (2). Although LCH presents with heterogeneous clinical manifestations based on the accumulation of mononuclear phagocytes in various tissues and organs (3), stomach involvement in LCH is very rare in children. Moreover, solid tumors of the stomach in children are rare (4, 5), leading pediatric oncologists and pathologists to be inexperienced in the diagnosis and treatment of tumors originating from the stomach in children. Herein, to the best of our knowledge, we report the first case of gastric LCH in a school-aged child in our hospital that was initially misdiagnosed as gastric lymphoma with recurrent abdominal pain.

## 2 Case description

A 9-year-old boy presented to our outpatient department with recurrent abdominal pain that had lasted 1 month. He experienced the abdominal pain mainly in the upper and middle abdominal regions. He did not experience hematochezia, melena, fever, night sweats, weight loss, or rashes. Physical examination revealed mild tenderness of the upper middle abdomen. No enlarged lymph nodes or hepatosplenomegaly were observed. He was admitted to a local general hospital, and gastroscopy revealed nodular erosive gastritis. A pathological biopsy revealed atypical cells in the esophagus and stomach. Furthermore, immunohistochemistry was positive for LCA and p53 and negative for HMB45, P40, P63, MPO, CD19, CD20 and CD117. The Ki-67 index was 80%. Finally, the patient was diagnosed with lymphoma by a local pathologist.

**Figure 1:**
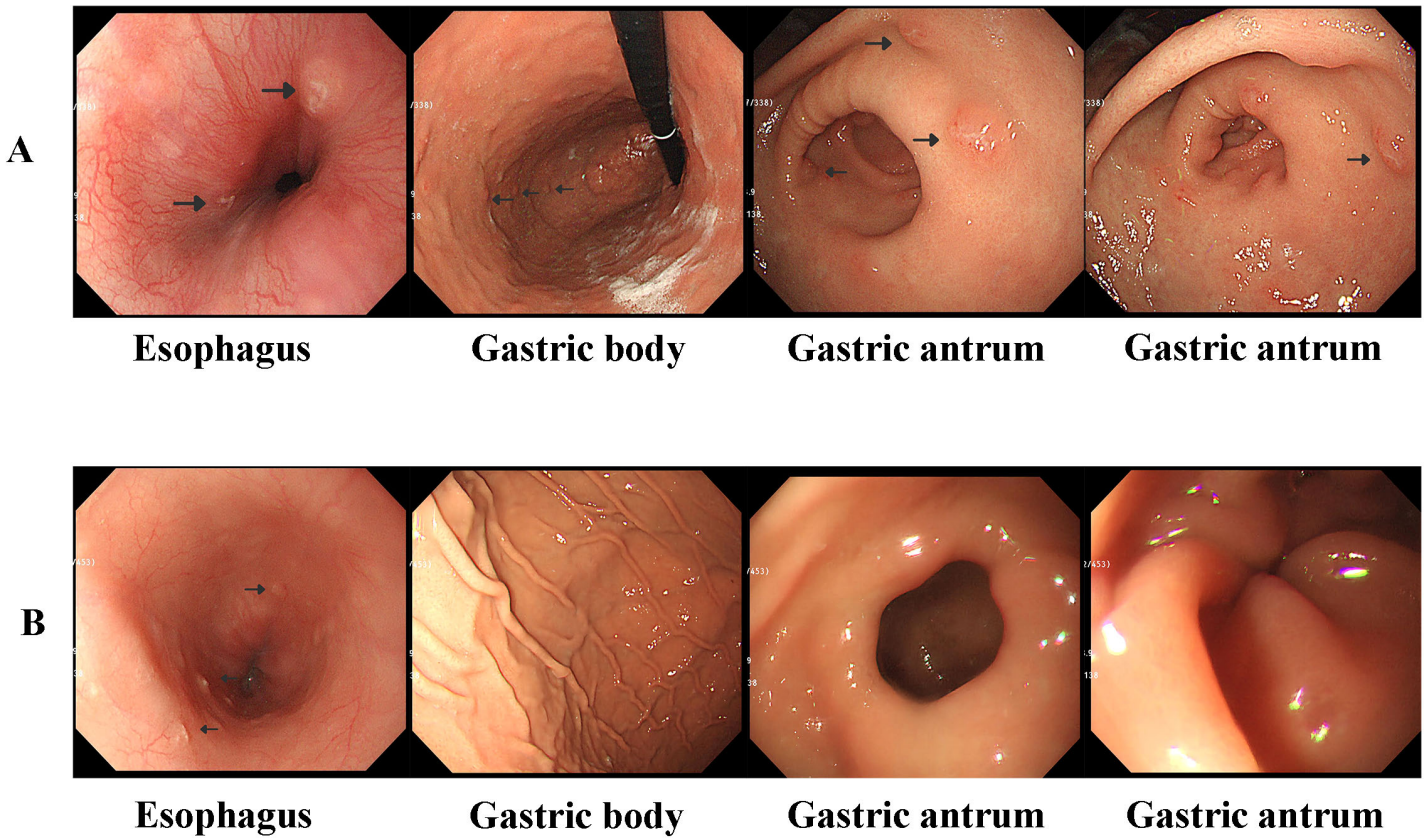
**(A)** Gastroscopy revealed localized esophageal erosions and gastric nodules and ulcers at diagnosis. **(B)** Gastroscopy revealed that the gastric nodules and ulcers disappeared after 6 weeks of induction chemotherapy; however, esophageal erosions still existed.

**Figure 2:**
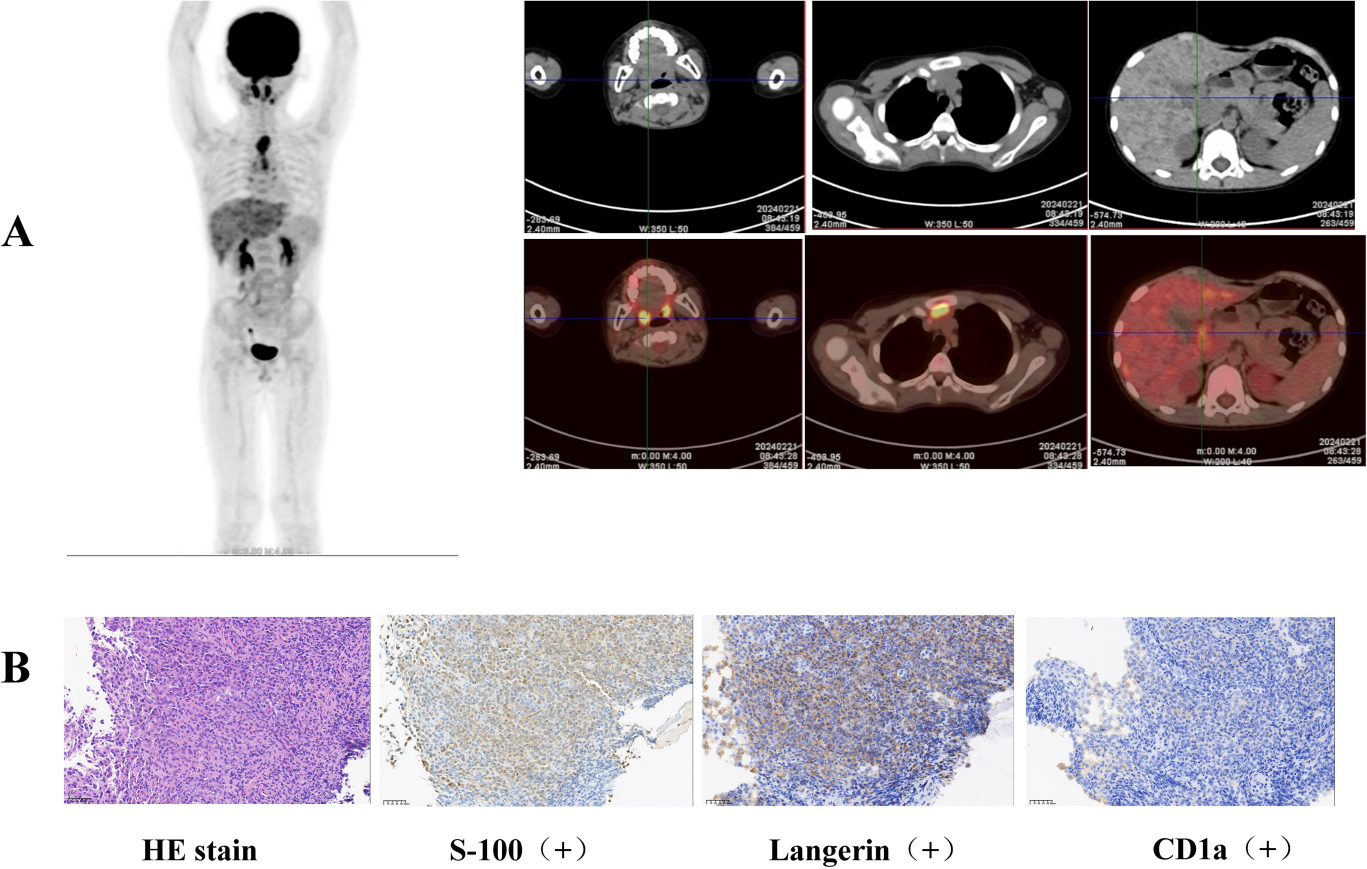
**(A)** Elevated FDG metabolism was detected in the gastric body, gastric antrum, nasopharynx, left submandibular gland, lymph nodes, thymus, and liver by whole-body FDG PET/CT examination. **(B)** Pathological biopsies revealed atypical cells in the gastric antrum expressing CD 1a, S-100, and Langerin.

The patient was referred to our hospital for further treatment. This was due to the rarity of gastric lymphoma during childhood. Due to the patient’s inability to acquire pathological biopsy tissue from the initial hospital in a timely manner, we opted to perform a repeat gastroscopy to obtain tissue for biopsy. We could still clearly observe gastric nodules and ulcers (**Figure 1A**). Pathological biopsies of the esophagus, descending duodenum, gastric antrum, gastric angle, and descending duodenum were performed. The results revealed atypical cells in the gastric antrum expressing CD 1a, S-100, and Langerin (**Figure 2B**). Furthermore, we detected 38 LCH-related genes, including *KRAS*, *ARAF*, *ERBB2*, *MAP2K1*, *ERBB3*, *MAP2K2*, and *BRAF*. Moreover, the patient harbored a deletion mutation in exon 12 of *BRAF*(c.1457_1471del), which led to an amino acid deficiency at positions 486 to 490.

## 3 Diagnostic assessment, therapeutic intervention, and follow-up

LCH was diagnosed based on the clinical manifestations, pathological biopsy, and genetic examination. Whole-body FDG PET/CT revealed elevated FDG metabolism in the gastric body, gastric antrum, nasopharynx, left submandibular gland, lymph nodes, thymus, and liver (**Figure 2A**). Therefore, the patient was diagnosed with high risk organ involvement (RO+) multisystem LCH. LCH III-directed induction chemotherapy (prednisone, 40mg/m^2^/d orally, day1-28, afterwards weekly reduction; vincristine, 2mg/m^2^/d, iv, bolus, day1, 8, 15, 22, 29, 36) was administered. Due to the unavailability of vinblastine in China, vincristine was utilized as a substitute. After 1 week of chemotherapy, the patient’s abdominal pain disappeared. After 6 weeks of induction chemotherapy, gastroscopy revealed that the number of lesions in the stomach and esophagus had significantly reduced (**Figure 1B**).

## 4 Discussion

LCH is a rare disease with an incidence of 3 to 9 cases per million children. As the largest pediatric cancer diagnosis and treatment center in Sichuan Province, China, our hospital has recorded 255 cases of LCH since 2013. However, the present case was the first patient with LCH and lesions in the stomach in our hospital. In theory, histiocytic proliferation and infiltration of LCH can affect any organ or system of the body; however, the skin, bone, liver, spleen, lymph nodes, lungs, and bone marrow are the most commonly involved organs in children. According to the literature review, there are sporadic reports of neonate or adult LCH with stomach involvement (6, 7). During the neonatal period, bloody stools, vomiting, and protein-losing enteropathy are the first signs of LCH with gastrointestinal tract involvement, which appear mostly in the first 4 weeks of life. Furthermore, skin eruptions are usually the initial symptoms, and at-risk organs (hematopoietic system, liver, and spleen) are often affected in newborns with LCH and gastrointestinal tract involvement (6). In contrast, lesions in adult LCH are localized to the stomach, and patients often have no symptoms (8, 9), dysphagia (7), or stomach discomfort (10). Unlike newborns and adults with gastric LCH, our patient presented with recurrent abdominal pain, and gastroscopy revealed nodular erosive gastritis. Similar to neonatal LCH, the patient was defined as having multisystem LCH with involvement of the stomach, nasopharynx, submandibular gland, lymph nodes, thymus, and liver. Notably, he had no skin lesions, which is a highlighted clinical feature compared with neonatal LCH.

Gastric tumors in pediatric populations are rare and prone to misdiagnosis due to the inexperience of pediatricians and pathologists. Gastrointestinal stromal tumors (11), lymphomas (12), gastric inflammatory myofibroblastic tumors (13), gastric synovial sarcomas (14), and LCH are known to occur in the stomach in children. It has been reported that the incidence of gastric lymphoma is 0.94% of all lymphomas, and the histopathological subtypes include Burkitt lymphoma, non-Burkitt mature B-cell lymphoma, anaplastic large cell lymphoma, marginal zone lymphoma (MZL), and the unspecified type (12). Our patient was initially diagnosed with lymphoma by a pathologist at a local general hospital. The boy had no fever, night sweats, weight loss, or lymph node enlargement. Furthermore, immunohistochemical stomach biopsy revealed no positive markers of B-cell origin (negative for CD19 and CD20). Therefore, we reconducted a biopsy of the gastric mucosa through gastroscopy to confirm the diagnosis. The pathologist in our hospital selected immunohistochemistry for CD1a, S-100, and Langerin protein molecules based on the morphological features and clinical manifestations of the patient. Finally, the patient was diagnosed with LCH, avoiding excessive chemotherapy owing to a misdiagnosis of lymphoma, with the help of the pathologist. In summary, the diagnosis of rare tumors in children relies on close communication between pediatricians and pathologists.

There is a significant difference in the prognosis of patients with gastric LCH between adults and children. In adults, gastric LCH often has a favorable prognosis with local resection or observation (7–10). In contrast, the prognosis of gastric LCH in newborns is poor. A previous study recorded 78.5% mortality, and 50% of the patients died within the first 4 months (6). A retrospective study also suggested that LCH in children with gastrointestinal tract involvement might be associated with a 4-fold risk for risk organ involvement and can decrease the survival rate, independent of organ involvement (15). Our patient responded well to LCH III-directed chemotherapy, and further follow-up is needed, especially to determine whether there is recurrence after discontinuation of the medication. Based on the *BRAF* deletion mutation, an oral BRAF inhibitor (dabrafenib) could be considered to further control the active disease.

In conclusion, although tumors in the stomach of children are rare, a differential diagnosis of gastric tumors should be established through multidisciplinary collaboration to avoid incorrect therapy. Unlike neonatal gastric LCH, LCH with stomach involvement in school-aged children is sensitive to chemotherapy and may have a good prognosis with prompt treatment.

## 5 Patient perspective

The patient’s parents provided their written informed consent to participate in this study. Written informed consent was obtained for the publication of this case report.

## Data Availability

The raw data supporting the conclusions of this article will be made available by the authors, without undue reservation.
